# Effect of resistance circuit training on comprehensive health indicators in older adults: a systematic review and meta-analysis

**DOI:** 10.1038/s41598-024-59386-9

**Published:** 2024-04-17

**Authors:** Chenxi Hu, Yunpeng Xia, Dongye Zeng, Mingyi Ye, Tao Mei

**Affiliations:** 1 Institute of Artificial Intelligence in Sports, Capital University of Physical Education and Sports, Beijing, 100191 China; 2 Department of Chinese Academy of Sport and Health, Beijing Sport University, Beijing, 100084 China; 3 School of Sports Medicine and Rehabilitation, Beijing Sport University, Beijing, 100084 China

**Keywords:** Resistance circuit training, Older adults, Body composition, Blood pressure, Functional autonomy, Cardiorespiratory endurance, Health care, Disease prevention, Geriatrics

## Abstract

The aging process leads to the degeneration of body structure and function. The objective of this study is to conduct a systematic review and meta-analysis of the effects of resistance circuit training (RCT) on comprehensive health indicators of older adults. PubMed, Embase, and Web of Science were searched until August 2023. Primary outcomes were body composition, muscle strength, cardiorespiratory endurance, blood pressure, and functional autonomy. Muscle function and exercise intensity subgroups were analyzed. RCT reduces body fat (MD = − 5.39 kg, 95% CI − 10.48 to − 0.29), BMI (MD = − 1.22, 95% CI − 2.17 to − 0.26), and body weight (MD = − 1.28 kg, 95% CI − 1.78 to − 0.78), and increases lean body mass (MD = 1.42 kg, 95% CI 0.83–2.01) in older adults. It improves upper limb strength (SMD = 2.09, 95% CI 1.7–2.48), lower limb strength (SMD = 2.03, 95% CI 1.56–2.51), cardiorespiratory endurance (MD = 94 m, 95% CI 25.69–162.67), and functional autonomy (MD = − 1.35, 95% CI − 1.73 to − 0.96). High-intensity RCT benefits BMI and body weight, while low-intensity exercise reduces blood pressure. RCT improves muscle function in push, pull, hip, and knee movements in older adults. RCT improves body composition, muscle strength, cardiorespiratory endurance, blood pressure, and functional autonomy in older adults. High-intensity training is superior for body composition, while moderate to low intensity training is more effective for lowering blood pressure.

## Introduction

Aging is an irreversible process characterized by the gradual deterioration of the body's functions and structures over time^1^. This results in the degeneration of the structure and function of the physiological systems of the human body, including reduced muscle strength, decreased flexibility, and a decline in maximum oxygen uptake. All of these factors can impact the quality of life and the functionality of older individuals^2–5^. Moreover, older adults in the early stages of age-related functional decline are particularly vulnerable to health risks^6^. One critical aspect of the health and functional capacity of older individuals involves changes in body weight and body composition during the aging process^7,8^. Typically, muscle mass decreases, and fat mass increases^9,10^. The reduction in muscle strength can result in sarcopenia, which raises the risk of falls and mortality^11–13^.

The World Health Organization (WHO) and the American College of Sports Medicine (ACSM) recommend that older adults engage in at least 150 min of moderate-intensity aerobic exercise per week or 75 min of vigorous-intensity exercise^2^. Currently, one of the most common training methods for older adults is circuit training^14–17^. This form of resistance circuit training efficiently targets both cardiorespiratory endurance and strength systems. Most importantly, RCT is a safe exercise method for older adults with high adherence^18^.

Randomized controlled trials have demonstrated that RCT can effectively improve various aspects of health, such as cardiorespiratory fitness, physical function, and body composition, in older adults^16,19–22^. High-intensity resistance circuit training, in particular, has been shown to increase muscle strength, muscle mass, and bone mineral density in older adults^23,24^. Moreover, RCT has been associated with a reduction in risk factors related to cardiovascular diseases in older individuals, such as lowering systolic and diastolic blood pressure^20,25^. A meta-analysis focused on RCT’s effects on muscle strength in older adults has indicated a more pronounced improvement in lower limb strength compared to upper limb strength. Furthermore, RCT has been shown to improve body composition and walking endurance in middle-aged and older women^18,26^.However, previous studies and meta-analyses have included both healthy and diseased populations, and have failed to differentiate between randomized controlled trials (RCTs) and single-arm studies. They have overlooked the effects of resistance circuit training (RCT) on blood pressure, cardiorespiratory fitness, and functional autonomy in older adults. To address this gap, our research presents the first systematic review and comprehensive meta-analysis to examine the impact of RCT on comprehensive health indicators in older adults, including muscle strength, cardiorespiratory endurance, functional autonomy, blood pressure, and body composition. Additionally, we assess the impacts of different intensities of resistance circuit training on key health indicators in the elderly.

## Methods

This meta-analysis adhered to PRISMA 2020 guidelines. To ensure transparency and rigor, this study has been registered in the PROSPERO database under the identifier CRD42023445449.

## Search strategy

To conduct a thorough examination of the comprehensive effects of circuit-based training on health indicators in older adults, a systematic search of studies was performed, from the inception of databases to August 10, 2023, among PubMed, Web of Science, and Embase. The search strategy involved the utilization of specific MESH terms and keywords, including “Circuit-Based,” “Exercise,” and “elderly people,” these terms were combined with the Boolean search terms “OR” and “AND” (refer to Supplementary Material A1. for the detailed search strategy). Two reviewers independently assessed the titles and abstracts. In cases of disagreement, a third reviewer made the final judgment for study selection.

### Inclusion and exclusion procedures

The PICOS principle was applied as inclusion criteria to select relevant studies:

Participants: The study included healthy and older adults.

Intervention: The mode of intervention in the study had to be resistance circuit training.

Control Group: The control group's intervention was no exercise.

Outcome Measures: The study considered outcome measures related to various health indicators in older adults.

Study Design: Only randomized controlled trials or controlled trials were included.

The following exclusion criteria were applied:

Participants: Studies involving animal models and populations with disease were excluded.

Publication Type: Abstracts, reviews, and conference articles were excluded.

Language: Studies published in languages other than English were excluded.

Data Completeness: Studies with incomplete reports of data were excluded.

Intervention: Studies involving the use of medications or supplements as part of the intervention were excluded.

### Data extraction

Two reviewers independently assessed titles, abstracts, and full texts of citations and extracted relevant data. Discrepancies were resolved through discussion, with a third reviewer assisting in reaching a consensus. WebPlotDigitizer was used to extract data from graphs, and authors were contacted for additional information if necessary. Extracted data included subject demographics, intervention details, exercise duration, and outcome measures. Data were presented as mean ± SD. In accordance with the ACSM position statement on physical activity and training intensity (Norton et al., 2010), eligible studies were classfied according to the research situation: 40% < maximal heart rate (HRmax) < 55%, 8 < Rating of Perceived Exertion (RPE) < 10, or 30% < 1RM < 50% were determined as light-intensity; 55% < HRmax < 70%, 11 < RPE < 13, or 50% < 1RM < 70% were considered to be of moderate-intensity; 70% < HRmax < 90%, 14 < RPE < 16, or > 70% 1RM were determined as high intensity^27^.This classification system aids in assessing and comparing intensity levels of resistance circuit training interventions and understanding their impact on health outcomes in older adults. Note that specific criteria may vary among studies, but this information aligns with ACSM guidelines.

### Quality assessment and certainty of evidence

Two investigators independently assessed the quality and risk of bias for each study using the risk of bias 2.0 (ROB2) tool. R software was utilized to visualize the risk of bias. In cases of disagreement between the two researchers, a third researcher was consulted.

The certainty of evidence was evaluated following the GRADE guidelines^28^. Evidence was downgraded if there were serious risks of bias, imprecision, inconsistency, indirectness, or publication bias. Outcome included body composition, upper limb muscle strength, lower limb muscle strength, cardiorespiratory endurance, functional autonomy, systolic blood pressure, and diastolic blood pressure. Each outcome was assigned one of four levels of evidence quality: high, moderate, low, or very low. Detailed evaluation results can be found in Supplementary Material A2.

### Statistical analysis

The raw data were entered into R (version 4.2.1) using the meta package for meta-analysis. Mean differences (MD) and 95% confidence intervals (CI) were calculated for outcomes measured on the same scale, while standardized mean differences (SMD) and 95% CIs were used for outcomes measured on different scales. Effect sizes were categorized as small (below 0.2), moderate (0.2–0.8), or large (exceeding 0.8) based on the Cochrane Handbook^29^. Heterogeneity was assessed using the I^2^ test and Cochrane's Q test, with I^2^ values indicating the degree of heterogeneity. Random-effects models were used for I^2^ > 50%, and fixed-effects models for I^2^ < 50%^30^. Subgroup analyses explored heterogeneity and clinical significance, focusing on different resistance circuit training (RCT) intensities' effects on body composition, blood pressure, and muscle functions. Forest plots visualized effect sizes and 95% CIs. The significance level was set at *P* < 0.05. Muscle strength subgroup analysis was conducted based on four categories: upper limb push, upper limb pull, lower limb hip dominant movement, and lower limb knee dominant movement^31^.

## Result

### Study selection and characteristics

**Figure 1:**
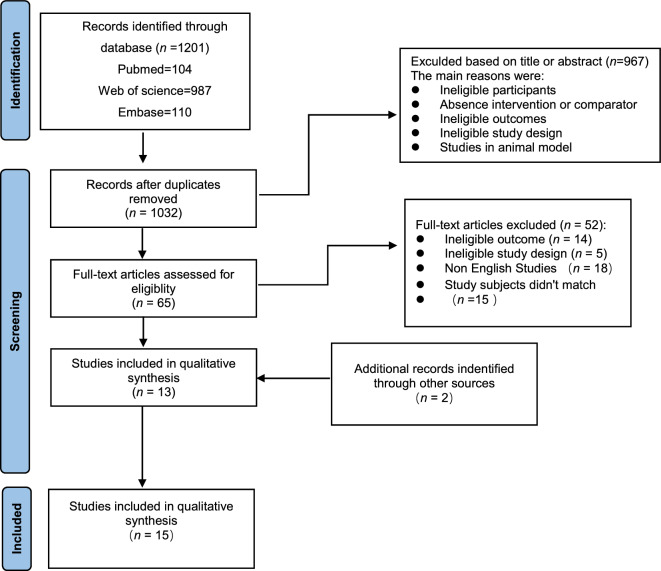
PRISMA flowchart of study selection.

A total of 1201 articles were initially identified through the search process. After removing 169 duplicate articles, a review of 1032 abstracts and titles was conducted, resulting in the exclusion of 968 articles. Ultimately, 15 articles met the inclusion criteria for this systematic review, and a meta-analysis was performed using the outcome measures from these articles (Fig. 1).

Fifteen studies were included, involving a total of 576 participants aged 60 to 75 years. Each study consisted of a RCT group and a blank control group. The intervention period ranged from 8 to 56 weeks, with training sessions occurring 1 to 3 times per week and each session lasting between 30 and 60 min. According to the intensity classification of ACSM, the RCT interventions in the included studies comprised high intensity (n = 10), moderate to low intensity (n = 4), and progression from low to high intensity (n = 1). Additionally, the included studies reported outcomes related to body composition (n = 8), muscle strength (n = 5), cardiorespiratory endurance (n = 2), blood pressure (n = 6), and functional autonomy (n = 3) (Table 1).

**Table 1:** Description of studies included in the meta-analysis.

Study	Group (n)	Age	Summary of Intervention			Outcomes
			Intensity (% RM or Other)	Duration (weeks/sessions per week/time)	Details (exercises/sets/repetitions)	
Marcos-Pardo et al. 2019	RCT-M (9)	69 ± 3.2	60–80% 1RM	12/3/-	6/-/8–12	BW, BMI, LM, GDLAM, Upper limb push, Knee function, Hip function
	RCT-F (15)			12/3/-	6/-/8–12	
	CG-M (9)	70 ± 4.1				
	CG-F (12)					
Bocalini et al. 2012	OW-RCT (14)	64 ± 4	70% HRR	12/3/50	12/-/-	BW, FM, LM, BMI
	OW-CG (9)	63 ± 2				
	O-RCT (9)	62 ± 2	70% HRR	12/3/50	12/-/-	
	O-CG (9)	62 ± 1				
Mazini Filho et al. 2018	RCT (34)	60–75	OMNI-RES RPE4–8	12/3/-	8/3/8–12	BM, BMI, 6 MW, Knee function, Upper limb pull
	CG (31)					
Suzuki et al. 2018	RCT (16)	> 60	70% 1RM	56/2/75	6/-/8–12	BW, BMI, Upper limb pull, 6 MW
	CG (15)					
Ballesta-García et al. 2020	HIICT (18)	67.8 ± 6.2	RPE 12–18	18/2/60	5–12/-/-	BMI, DBP, SBP
	MICT (18)		RPE 6–14	18/2/60	5–12/-/-	
	CG (18)					
Choi et al. 2020	RCT (15)	75.1 ± 1.4	RPE 12–14	12/3/30	19/-/10–12	DBP, SBP
	CG (12)	72.3 ± 1.4				
Romero-Arenas et al. 2013	RCT (16)	61.6 ± 5.3	50–100% 6RM	12/2/35–47	6/1–3/6–12	FM, LM
	CG (7)					
Lee et al. 2018	RCT (12)	71.13 ± 2.75	RPE 11–14	8/3/50	8/3/12–15	DBP, SBP
	CG (12)	72.13 ± 2.72				
Ramos et al. 2022	RCT (23)	64.7 ± 6.74	OMNI-RES RPE 3–8	16/2/50	11/3/12–14	Upper limb pull, Knee function, GDLAM
	CG (22)	64.81 ± 4.34				
Rhodes et al. 2000	RCT (20)	68.8 ± 3.2	75% 1RM	52/2–3/60	8/3/8	BW, Upper limb push, Upper limb pull, Knee function
	CG (18)	68.2 ± 3.5				
Mirua et al. 2008	1 DW (29)	69.0 ± 6.5	70.3%-75.4% HRmax	12/1/40	6–8/3–5/15–20	BW, BMI, DBP, SBP, Upper limb pull
	2 DW (25)	69.5 ± 7.0		12/2/40	6–8/3–5/15–20	
	CG (23)	68.9 ± 7.5				
Min Fang et al. 2020	RCT (12)	70.8 ± 5.8	40–65% 1RM	8/3/45	10/1–3/10–12	DBP, SBP
	CG (8)	71.8 ± 4.8				
Juan Zhang et al. 2021	RCT (11)	70.3 ± 5.7	40–65% 1RM	8/3/45	10/1–3/10–12	DBP, SBP
	CG (7)	71.6 ± 5.2				
Ballesta-García et al. 2019	HIICT (18)	67.8 ± 6.2	RPE 12–18	18/2/60	5–12/-/-	6 MW, BMI
	MICT (18)		RPE 6–14	18/2/60	5–12/-/-	
	CG (18)					
Pyka et al. 1994	RCT (8)	68.2 ± 1	65–75% 1RM	52/3/60	12/3/8	Upper limb push, Upper limb pull Knee function Hip function
	CG (6)					

*"-" stands for missing data.n number, RCT resistance circuit training, CG control group, OW over weight,O obesity, HIICT high intensity interval resistance circuit training, 1DW one day a week, 2DW two day a week, MICT moderate intensity continuous training, 1RM One Rep Max, F female, M male, HRR Heart Rate Reserve, RPE Rating of Perceived Exertion, HRmax Heart Rate Max, BW body weight, BMI body mass index, FM fat mass, LM lean mass, GDLAM Heart Rate score , 6 MW 6 min walking, DBP diastolic blood pressure, SBP systolic blood pressure.

### Risk of bias of included studies

Ten of the 15 studies included had some concerns, three were High risk, Two were Low risk, and the major bias was in the areas of Randomization process and Deviations from intended interventions. It is low risk in the Measurement of the outcome and Selection of the reported field. Detailed risk of bias assessment results are provided in Supplementary Material A3.

## Results of the meta-analysis

### Body composition

**Figure 2:**
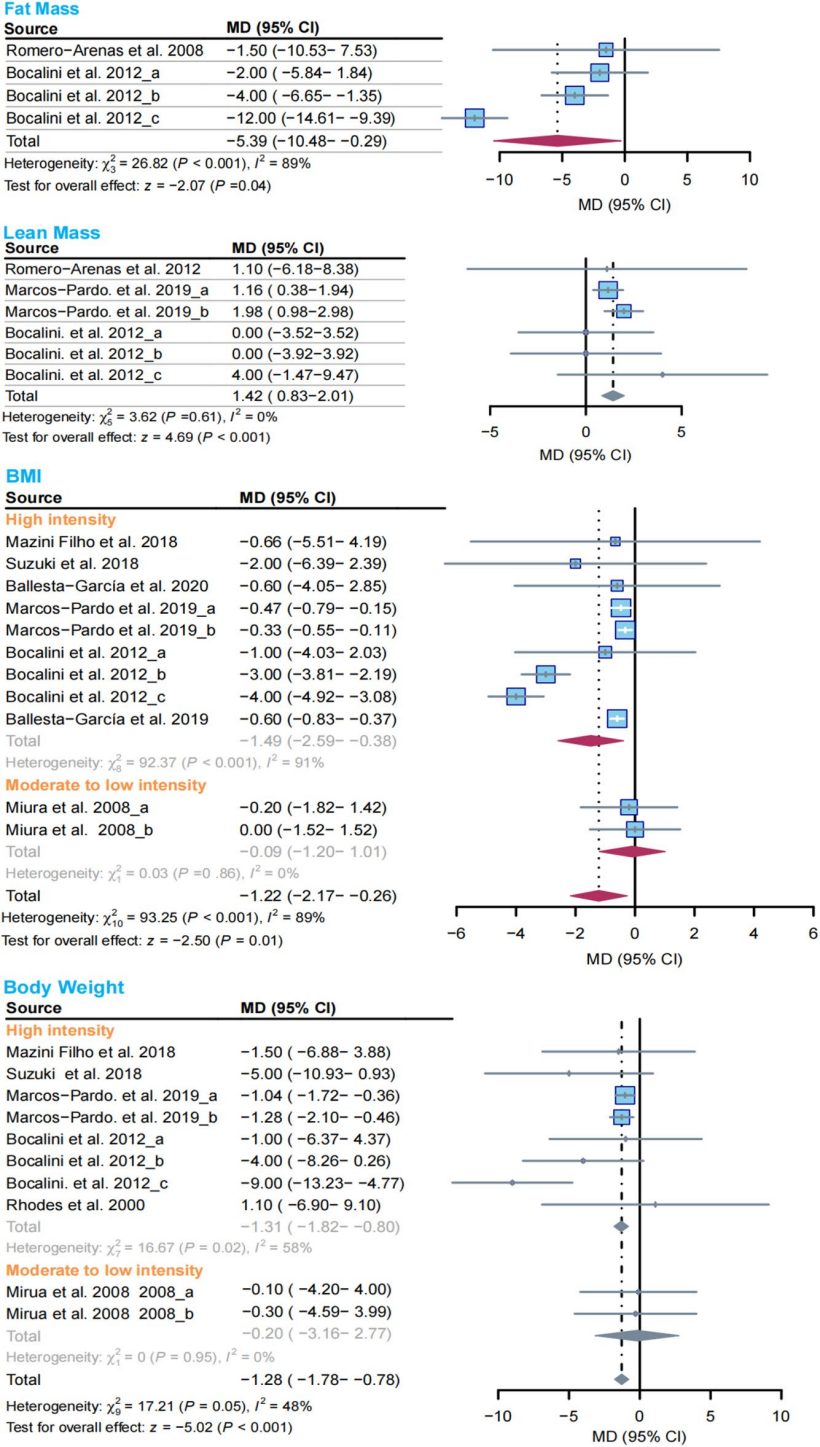
Effect of RCT on body composition in older adults. (**a**), (**b**), and (**c**) in the figure represent different populations in the same study, diamonds in the forest plot represent the overall effect size. Gray represents the results calculated using the fixed effects model, and red represents the random effects model.

**Figure 3:**
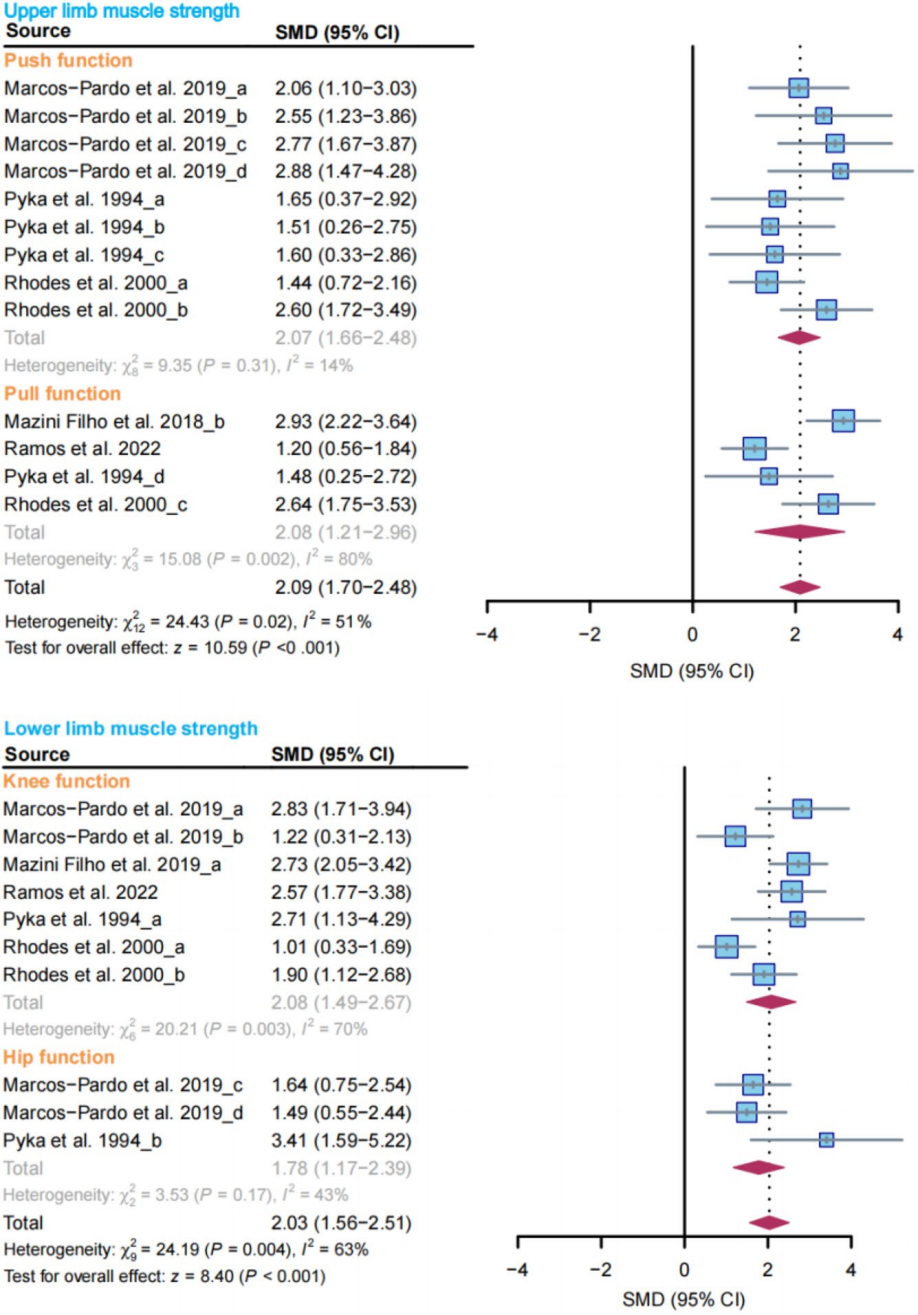
Effect of RCT on muscle strength in older adults. (**a**), (**b**), (**c**) and ‘d’ in the figure represent different populations or different body parts in the same study, diamonds in the forest plot represent the overall effect size, and red indicates the results calculated using the random effects model.

**Figure 4:**
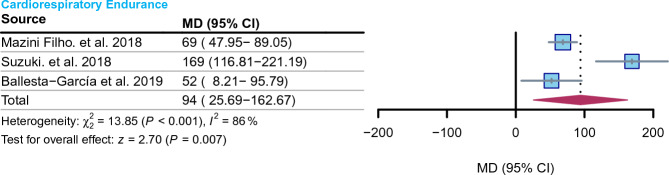
Effect of RCT on cardiorespiratory endurance in elderly individuals. Diamonds in the forest plot represent the overall effect size, and red indicates the results calculated using the random effects model.

After analyzing the data from the included studies that assessed body composition as an outcome, we found that compared to the control group, the RCT group showed significant effects on decreasing fat mass (MD =- − 5.39 kg, 95% CI − 10.48 to − 0.29, I^2^ = 89%, *P* = 0.04), BMI (MD = − 1.22, 95% CI − 2.17 to − 0.26, I^2^ = 89%, *P* = 0.01) (Fig. 3), body weight (MD = − 1.28 kg, 95% CI − 1.78 to − 0.78, I^2^ = 48%, *P* < 0.001) (Fig. 4) and increasing lean mass (MD = 1.42 kg, 95% CI 0.83–2.01, I^2^ = 0%, *P* < 0.001) in older adults (Fig. 2).

Subgroup analyses revealed that high-intensity RCT reduced BMI (MD = − 1.49, 95% CI − 2.59 to − 0.38, I^2^ = 91%) and body weight (MD = − 1.31 kg, 95% CI − 1.82 to − 0.8, I^2^ = 58%). However, no significant changes in BMI or body weight were found with low- and moderate-intensity RCT (Fig. 2).

### Muscle strength

The meta-analysis of the seven included studies of muscle strength revealed a improvement in upper limb strength function among older adults (SMD = 2.09, 95% CI 1.7–2.48, I^2^ = 51%, *P* < 0.001). Subgroup analyses based on basic body movement patterns indicated that RCT enhanced both upper limb push function (SMD = 2.07, 95% CI 1.66–2.48, I^2^ = 14%) and upper limb pull function in older adults (SMD = 2.08, 95% CI 1.21–2.96, I^2^ = 80%).

A subgroup analysis of the five included studies demonstrated that RCT improved knee function (SMD = 2.08, 95% CI 1.49–2.67, I^2^ = 70%) and hip function (SMD = 1.78, 95% CI 1.17–2.39, I^2^ = 43%). The overall effect size for lower limb strength function showed improvement (SMD = 2.03, 95% CI 1.56–2.51, I^2^ = 63%, *P* < 0.001) (Fig. 3).

### Cardiorespiratory endurance

The meta-analysis findings suggested that RCT may enhance the 6-min walking distance of older adults (MD = 94 m, 95% CI 25.69–162.67, I^2^ = 86%, *P* = 0.007) (Fig. 4).

### Blood pressure

The effect of RCT on blood pressure in the elderly was examined in the meta-analysis of the six included studies. The results showed a difference between the RCT and control groups in terms of systolic blood pressure (SBP) (MD = -9.8 mmHg; 95% CI − 13.26 to − 6.31, I^2^ = 44%, *P* < 0.001). Similar changes were observed in diastolic blood pressure (DBP) (MD = − 4.4 mmHg; 95% CI − 6.59 to − 2.15, I^2^ = 6%, *P* < 0.001).

**Figure 5:**
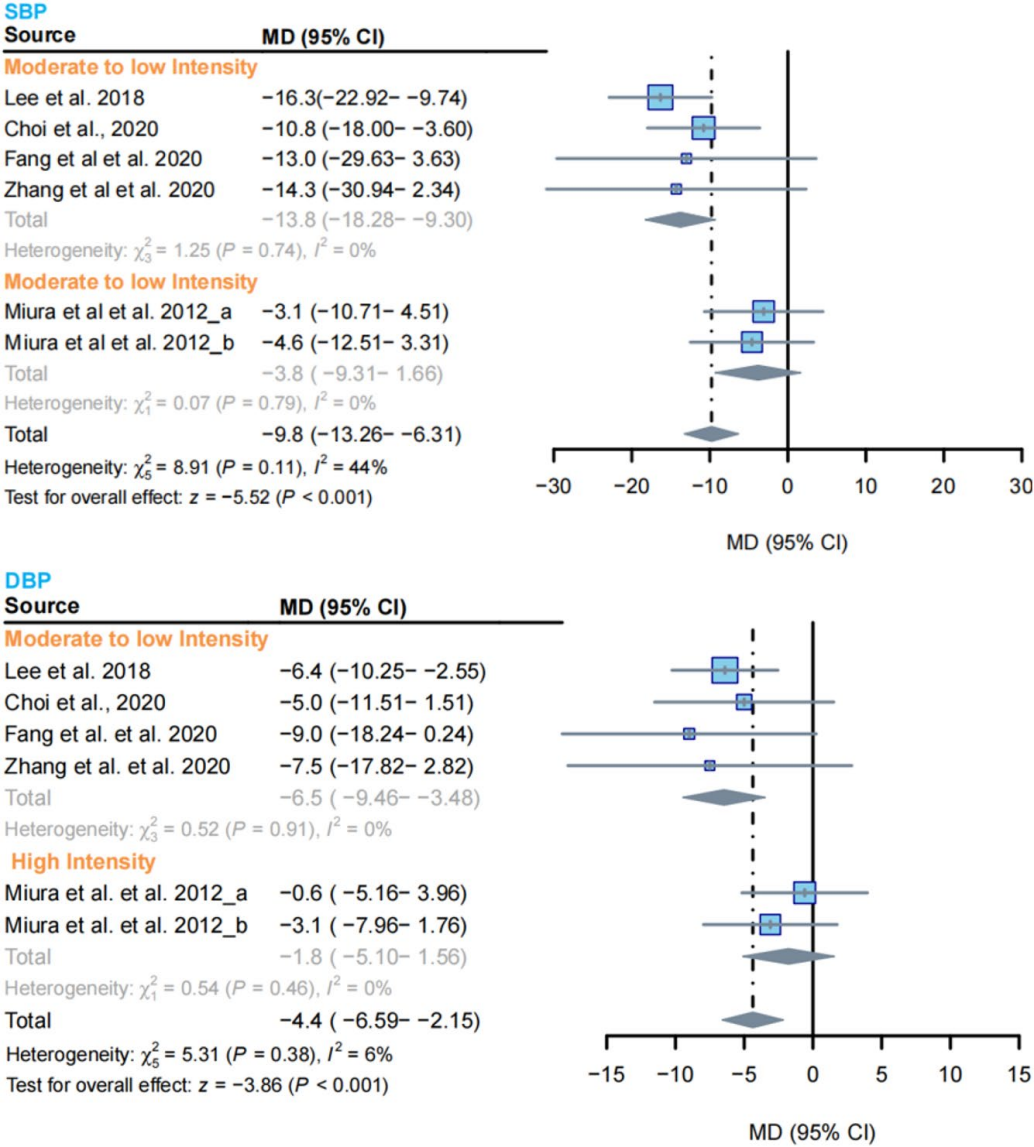
Investigation of the Impact of RCT on Blood Pressure in Older Adults: In the forest plot, diamonds represent the overall effect size. Gray shading indicates results calculated using the random effects model. SBP refers to systolic blood pressure, while DBP refers to diastolic blood pressure. The labels (**a**) and (**b**) in the figure represent different populations within the same study.

Subgroup analysis was conducted based on exercise intensity. It was found that SBP was lower in moderate to low intensity exercise (MD = − 13.8 mmHg; 95% CI − 18.28 to − 9.3, I^2^ = 0%), while no changes were observed with high-intensity exercise. Similarly, modest to low intensity exercise was associated with a decrease in DBP (MD = − 6.8 mmHg; 95% CI − 9.46 to − 3.48, I^2^ = 0%), whereas high-intensity exercise did not show changes (Fig. 5).

### Functional autonomy

**Figure 6:**
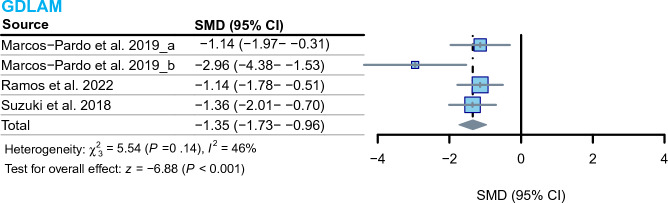
Effect of RCT on functional autonomy in elderly individuals. Diamonds in the forest plot represent the overall effect size, and gray indicates the results calculated using the random effects model. Latin American Development Group for Maturity (GDLAM), where lower GDLAM scores indicate better functional autonomy in older individuals. (**a**) and (**b**) in the figure represent different populations in the same study.

The meta-analysis of the three included studies that reported functional autonomy demonstrated that RCT interventions led to an improvement among older adults. The assessment of functional autonomy was performed using the GDLAM index in these studies. The results indicated a decrease in the GDLAM index after the RCT intervention compared to the control group (MD = − 1.35, 95% CI − 1.73 to − 0.96, I^2^ = 46%, *P* < 0.001) (Fig. 6).

## Discussion

This meta-analysis comprehensively examined the impact of resistance circuit training on various health indicators in older adults, including body composition, muscle strength, endurance, blood pressure, and functional autonomy. Subgroup analyses identified movement patterns and exercise intensity as sources of heterogeneity. The findings from 15 studies demonstrate the positive effects of resistance circuit training in older adults: 1. Resistance circuit training effectively reduces body weight, BMI, and fat mass while promoting lean body mass, particularly with high-intensity regimens. 2. Muscle strength in both upper and lower limbs significantly improves with resistance circuit training. 3. Resistance circuit training enhances cardiorespiratory endurance, as indicated by improved 6-min walking performance. 4. It effectively lowers blood pressure, with medium- to low-intensity programs being more advantageous. 5. Resistance circuit training improves functional autonomy in older people.

This study’s findings align with previous research on body composition, which reveals that resistance circuit training is an effective means to enhance lean body mass and reduce fat mass in older adults^32^. With advancing age, older adults often experience a decline in muscle mass and an increase in fat mass. The decline in muscle mass can lead to mobility issues, reduced physical strength, and balance problems, potentially increasing the risk of falls and fractures^33^. Increased fat mass, on the other hand, may elevate the risk of chronic diseases such as diabetes, hypertension, and cardiovascular issues^34^. Resistance circuit training has played a positive role in improving body composition among various populations. Resistance circuit training has been found to have a similar effect as aerobic training on reducing body fat mass in postmenopausal women, with reductions in total body fat, subcutaneous fat, and abdominal fat observed after low-intensity resistance circuit training, alongside reductions in blood pressure^35^. In Pablo et al. study, a 12-weeks resistance circuit training program improved lean body mass, reduced fat mass, and enhanced functional autonomy in older adults aged between 65 and 75 years^36^. Another study found that resistance circuit training yielded similar results to traditional strength training in improving lean body mass while also reducing fat mass^21^. Resistance circuit training typically combines resistance exercises (such as weightlifting, push-ups, squats, etc.) with aerobic exercises (e.g., jumping jacks, running, jump rope, etc.). Resistance exercises contribute to minor muscle tissue damage and subsequent reconstruction, leading to increased muscle mass^37,38^, while aerobic exercises increase energy expenditure, aiding in reducing fat mass^39^. This could be a contributing factor to the beneficial effects of resistance circuit training on body composition.

Weight is the dynamic result of muscle, fat, and water balance within the body. Consistent with prior research, this study also found significant effects of resistance circuit training on weight and BMI in older adults. In Ramos-Campo's research, resistance circuit training significantly reduced the weight (average decrease of 1.6 kg) and body mass index (average decrease of − 0.70 kg/m^2^) of middle-aged and older women (aged 54–73 years)^32^. Another study discovered that resistance circuit training had significant intervention effects on weight (average decrease of − 3.81 kg) and body mass index (average decrease of − 1.77 kg/m2) in healthy adults (above 18 years old). Subgroup analysis revealed that the intervention effects were significant only in obese or overweight participants (obese: WMD = − 5.15 kg, 95% CI − 8.81 to − 1.50; overweight: WMD = − 3.89 kg, 95% CI − 7.00 to − 0.77), with no significant effect observed in normal-weight participants^40^. Subgroup analysis in this study indicates that high-intensity resistance circuit training is more conducive to weight and BMI reduction. This is likely attributed to the higher calorie expenditure and increased metabolic rate associated with higher intensity. Studies focused on older adults have demonstrated that aerobic combined with resistance training plans can increase metabolic rate and improve body composition. However, long-term training in older women can lead to metabolic adaptation and a decrease in metabolic rate^41^. This suggests that the optimal intensity for weight loss may vary due to individual differences, such as age. The suitability of high-intensity resistance circuit training for older adults should be determined based on their health status and physical fitness levels.

This study asserts that resistance circuit training is advantageous for enhancing muscle strength in older adults, aligning with previous research^18,32^. In postmenopausal older women, resistance circuit training has demonstrated moderate to large favorable effects on the strength of the arms (SMD = 0.81, 95% CI 0.34–1.28), trunk (SMD = 1.61, 95% CI 0.95–2.28), and lower limbs (SMD = 1.30, 95% CI 0.72–1.88)^32^. Among middle-aged and older participants (aged 55–74 years), resistance circuit training resulted in a 1.14 kg increase in upper body strength (95% CI 0.28–2.00) and an 11.99 kg increase in lower body strength (95% CI 2.92–21.06)^18^. Subgroup analysis in this study reveals that resistance circuit training has a positive impact on increasing upper-body pushing, upper-body pulling, knee flexor–extensor strength, and hip flexor–extensor strength in older adults (SMD: 1.78–2.08). This suggests that resistance circuit training's benefits on muscle strength are holistic, promoting overall health maintenance in older individuals. The ability of resistance circuit training to enhance muscle strength is attributed to its characteristics of high intensity, variety, overload training, and consistency. These elements collectively enhance muscle adaptability, resulting in increased strength.

Cardiorespiratory endurance is of paramount importance for older adults in maintaining physical health, enhancing quality of life, reducing the risk of chronic diseases, and decelerating the aging process. This study found that resistance circuit training can significantly enhance 6-min walking performance, a critical measurement of cardiorespiratory endurance, in older individuals. An 8-month aerobic training intervention effectively improved the 6-min walking performance in older adults, while resistance training demonstrated an improvement trend in the 6-min walking test without reaching statistical significance^42^. Nevertheless, a majority of studies still indicate the beneficial effects of resistance training on the 6-min walking test^43,44^. Maximal oxygen consumption (VO_2_ max) is another crucial indicator of cardiorespiratory endurance in older individuals.However, in this study, only one study reported improvements in VO_2_ max in older adults following resistance circuit training^21^, making it challenging to conduct a comprehensive meta-analysis.

Blood pressure levels in older adults serve as an indicator of cardiovascular health. Controlling blood pressure in older individuals is crucial for preventing and managing various chronic diseases and maintaining overall well-being. In this study, resistance circuit training demonstrated significant improvements in both systolic and diastolic blood pressure levels in older adults. The improvement in blood pressure through resistance circuit training may be attributed to the enhancement of vascular endothelial function. Multiple studies have found that resistance circuit training can effectively enhance vascular health indicators such as intima-media thickness (IMT) of the carotid artery, flow-mediated dilation (FMD) of the brachial artery, and pulse pressure (PP)^45,46^. Additionally, resistance circuit training may reduce the burden on the cardiovascular system by reducing body weight and fat mass, leading to a decrease in blood pressure.

Functional autonomy refers to an individual's ability to independently perform daily tasks and activities of daily living, encompassing physical independence, mobility, and the capacity to carry out routine tasks and activities. Functional autonomy is primarily assessed using the Latin American Development Group for Maturity (GDLAM), where lower GDLAM scores indicate better functional autonomy in older individuals^47^. This study found that resistance circuit training can effectively reduce GDLAM scores in older adults, thereby enhancing their functional autonomy. Among the included studies, high-intensity circuit resistance training has demonstrated a positive impact on functional autonomy, with significant reductions in GDLAM scores in the experimental groups^16,48,49^.

## Limitations

Our meta-analysis has limitations. Firstly, we included 2 controlled experiments due to limited research on resistance circuit training in older adults. Secondly, high heterogeneity remained in muscle function and body composition outcomes. Subgroup analyses based on gender and age were not feasible. A standardized criterion for measuring exercise intensity in older adults was lacking. Intervention periods ranged from 8 to 56 weeks, requiring cautious interpretation. Additionally, included studies had a high risk of bias and low overall quality.

Another notable limitation of our study is the reliance on a single study for analyzing the effects of high-intensity circuit training on blood pressure in older adults. This limited data source constrains the statistical robustness and generalizability of our findings within this specific subgroup. While the investigation into high-intensity training is driven by its potential relevance for older adults' health, the conclusions drawn should be interpreted with caution due to the small sample size.

## Conclusion

Resistance circuit training has positive effects in older adults, reducing fat mass and increasing lean body mass, especially with high-intensity training. Muscle function, including upper and lower limb strength, improves, along with knee and hip joint muscle function. Circuit training enhances cardiorespiratory endurance, as seen in improved 6-min walking distance. It also maintains blood pressure, particularly systolic, in moderate to low intensity. Functional autonomy improves using the GDLAM index in older adults.

### Supplementary Information

Supplementary Information 1.

Supplementary Information 2.

Supplementary Information 3.

## Data Availability

The datasets used or analyzed during the current study are available from the corresponding author upon reasonable request.
