# Supplementary material for: Effect of resistance circuit training on comprehensive health indicators in older adults: a systematic review and meta-analysis
Source: Sci Rep. 2024 Apr 17;14:8823. doi: 10.1038/s41598-024-59386-9 (PMC11021536; doi:10.1038/s41598-024-59386-9)
Supplement: Supplementary file 1 — Supplementary Information 1. [file 41598_2024_59386_MOESM1_ESM.docx]

**Supplementary Material A1：Search strategy**

**Pubmed**

#1: (Aged[Mesh]) OR (Elderly[Title/Abstract]) OR (older[Title/Abstract]) OR (Geriatric[Title/Abstract]) OR (Senior[Title/Abstract])

#2: (Circuit-Based Exercise[Mesh]) OR (Circuit-Based Exercises[Title/Abstract]) OR (Exercise, Circuit-Based[Title/Abstract]) OR (Exercises, Circuit-Based[Title/Abstract]) OR (Circuit Training[Title/Abstract]) OR (Training, Circuit[Title/Abstract]) OR (circuit resistance training[Title/Abstract]) OR (circuit weight training[Title/Abstract]) OR (circuit strength training[Title/Abstract])

#3: (randomized controlled trial[pt] OR controlled clinical trial[pt] OR randomized[tiab] OR placebo[tiab] OR drug therapy[sh] OR randomly[tiab] OR trial[tiab] OR groups[tiab])

#1 AND #2 AND #3

**Web of Science**

#1: (TS=(Circuit-Based Exercise) OR TS=(Circuit-Based Exercises) OR TS=(Exercise, Circuit-Based) OR TS=(Exercises, Circuit-Based) OR TS=(Circuit Training) OR TS=(Training, Circuit) OR TS=(circuit resistance training) OR TS=(circuit weight training) OR TS=(circuit strength training))

#2: (TS=(Aged) OR TS=(Elderly) OR TS=(older) OR TS=(Geriatric) OR TS=(Senior))

#3:(TS=(randomized controlled trial) OR TS=(controlled clinical trial) OR TS=(randomized) OR TS=(placebo) OR TS=(drug therapy) OR TS=(randomly) OR TS=(trial) OR TS=(groups))

#1 AND #2 AND #3

**EMBASE**

#1: aged:ti,ab,kw OR older:ti,ab,kw OR geriatric:ti,ab,kw OR senior:ti,ab,kw

#2: 'circuit-based exercise':ti,ab,kw OR 'circuit-based exercises':ti,ab,kw OR 'exercise, circuit-based':ti,ab,kw OR 'exercises, circuit-based':ti,ab,kw OR 'circuit training':ti,ab,kw OR 'training, circuit':ti,ab,kw OR 'circuit resistance training':ti,ab,kw OR 'circuit weight training':ti,ab,kw OR 'circuit strength training':ti,ab,kw

#3: 'randomized controlled trial':ti,ab,kw OR 'controlled clinical trial':ti,ab,kw OR randomized:ti,ab,kw OR placebo:ti,ab,kw OR 'drug therapy':ti,ab,kw OR randomly:ti,ab,kw OR trial:ti,ab,kw OR groups:ti,ab,kw

#1 AND #2 AND #3
