# Supplementary material for: Effect of resistance circuit training on comprehensive health indicators in older adults: a systematic review and meta-analysis
Source: Sci Rep. 2024 Apr 17;14:8823. doi: 10.1038/s41598-024-59386-9 (PMC11021536; doi:10.1038/s41598-024-59386-9)

Supplementary Material A3:Risk of bias of included studies

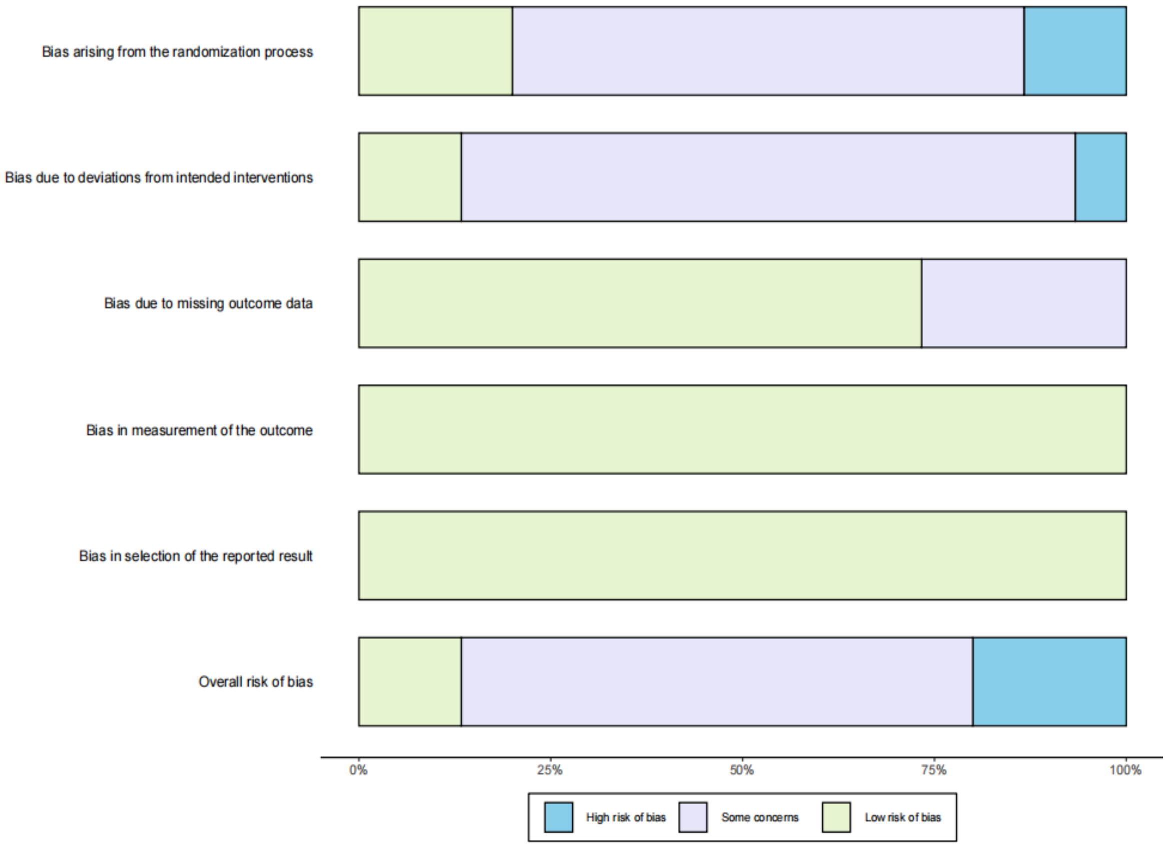

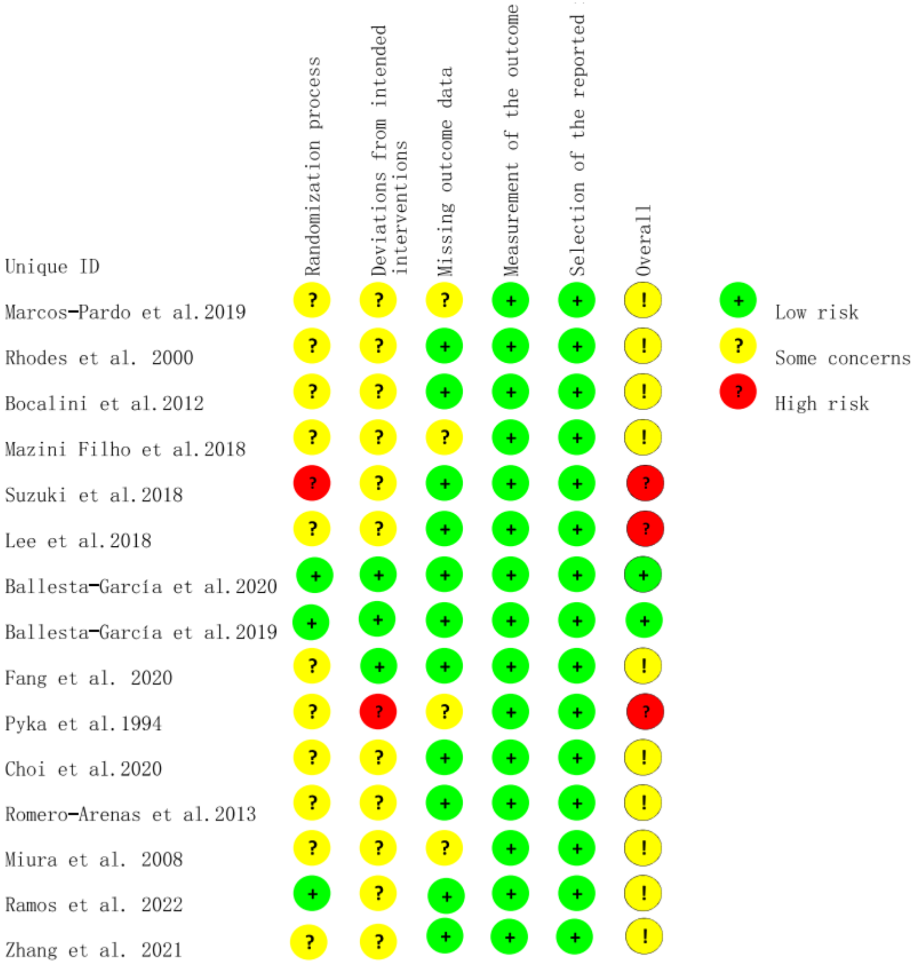

Supplement: Supplementary file 3 — Supplementary Information 3. [file 41598_2024_59386_MOESM3_ESM.pdf]
